# Multicellular Computing Using Conjugation for Wiring

**DOI:** 10.1371/journal.pone.0065986

**Published:** 2013-06-20

**Authors:** Angel Goñi-Moreno, Martyn Amos, Fernando de la Cruz

**Affiliations:** 1 Systems Biology Program, Centro Nacional de Biotecnología CSIC, Cantoblanco-Madrid, Spain; 2 School of Computing, Mathematics and Digital Technology, Manchester Metropolitan University, Manchester, United Kingdom; 3 Departamento de Biología Molecular e Instituto de Biomedicina y Biotecnología de Cantabria (IBBTEC), Universidad de Cantabria-CSIC-SODERCAN, Santander, Spain; Center for Genomic Regulation, Spain

## Abstract

Recent efforts in synthetic biology have focussed on the implementation of logical functions within living cells. One aim is to facilitate both internal “re-programming” and external control of cells, with potential applications in a wide range of domains. However, fundamental limitations on the degree to which single cells may be re-engineered have led to a growth of interest in multicellular systems, in which a “computation” is distributed over a number of different cell types, in a manner analogous to modern computer networks. Within this model, individual cell type perform specific sub-tasks, the results of which are then communicated to other cell types for further processing. The manner in which outputs are communicated is therefore of great significance to the overall success of such a scheme. Previous experiments in distributed cellular computation have used global communication schemes, such as quorum sensing (QS), to implement the “wiring” between cell types. While useful, this method lacks specificity, and limits the amount of information that may be transferred at any one time. We propose an alternative scheme, based on specific cell-cell conjugation. This mechanism allows for the direct transfer of genetic information between bacteria, via circular DNA strands known as plasmids. We design a multi-cellular population that is able to compute, in a distributed fashion, a Boolean XOR function. Through this, we describe a general scheme for distributed logic that works by mixing different strains in a single population; this constitutes an important advantage of our novel approach. Importantly, the amount of genetic information exchanged through conjugation is significantly higher than the amount possible through QS-based communication. We provide full computational modelling and simulation results, using deterministic, stochastic and spatially-explicit methods. These simulations explore the behaviour of one possible conjugation-wired cellular computing system under different conditions, and provide baseline information for future laboratory implementations.

## Introduction

The growing field of synthetic biology [1]–[4] is concerned with the application of engineering principles, concepts and techniques to the modification and/or construction of biological systems. This (re-)engineering may be motivated by a desire to better understand the underlying biological substrate, or by novel applications of biological “devices”. Although the field traces its roots back to early work on genetic engineering, it emerged as a serious research area at the turn of the century, with the simultaneous publication of two significant papers. The first, by Elowitz and Leibler [5], described a fully-synthetic genetic oscillator engineered into the *E. coli* bacterium. Their “repressilator” relied on mutual repression of a “loop” of connected genes in order to achieve oscillation. The other paper, due to Gardner, Cantor and Collins [6], outlined the design and construction of a synthetic toggle switch (also in *E. coli*), the state of which could be “flipped” from outside by either chemical or thermal induction. Both of these constructions are now standard motifs in the design of synthetic biological systems, and provided inspiration for the construction of a number of genetic devices [7]–[9]. However, just as the pioneers of computer technology quickly incorporated the early transistor into larger circuits in order to build the first solid-state computers, researchers in synthetic biology rapidly sought to build ever larger devices using these gene-based components.

There exists, though, a fundamental limitation on the amount and type of novel genetic “circuitry” that may be introduced into a single living cell. As the authors of [10] argue, “…establishing the wiring of an electronic circuit just requires linking each pair of connected elements by a wire (e.g. a piece of copper). But inside a cell, the cables need to have a different implementation: different proteins must be used for each different pair. Additionally, because of the intrinsic difficulties of implementing them, the resulting constructs are usually specific for the given problem and cannot be reused afterwards”. Because of these related issues of cross-talk and lack of modularity, many researchers now seek an alternative approach. By expanding the scope of synthetic biology beyond *single-cell* solutions, and into the domain of *multicellular* systems, we seek to harness the inherent power of biological “nanotechnology”, but in a way that readily allows for scalability, noise tolerance and component reusability. With this in mind, attention is turning to the engineering of microbial *consortia* [11]; *multiple* populations of microbes that can interact to perform functions beyond those achievable by *individual* populations. The power of such consortia is derived from both their *robustness* and their ability to perform complex tasks in a *distributed* fashion. These attributes are due to two main features; *communication* between consortium members (that is, the exchange of information between individuals), and *division of labour* (the overall behaviour of the system is the result of combining many sub-tasks, each performed by individuals or sub-populations) [11].

Recently, the notion of distributed, multicellular computation using engineered cells has gained increasing traction [10]. One of the first such systems was demonstrated by Basu *et al.* in 2005 [12], in which the authors demonstrated programmed pattern formation, using populations of engineered “sender” and “receiver” cells. More recently, Tamsir *et al.* [13] showed how simple logic gates may be constructed by programming communication between bacterial colonies, and Regot *et al.* [14] demonstrated a similar system in yeast. Central to the implementation of multicellular computation is controlled *communication* between cells and populations of cells. So far, this has *generally* been implemented using the *global* communication capabilities offered by *quorum sensing* (QS) [13]–[15] (although the use of bacteriophage has also recently been proposed [16]). Within the QS system, one cell (sender) uses small signalling molecules that diffuse over distance and thus reach other cells (receivers) [17]. Apart from the implementation of logical functions, QS has been used for other purposes, such as the synchronization of engineered oscillators [18]. However, we believe that multicellular computation will greatly benefit from a more *varied* range of communication protocols. As Ortiz and Endy highlight [18], QS-based communication is limited by both the *type* and *content* of messages that are possible using chemical signals.

We therefore propose a scheme for multicellular computation based on a *local* communication protocol. The foundation for this is the process of genetic exchange between bacteria known as *bacterial conjugation* [19], which has often been likened to “bacterial sex” [20], [21]. During conjugation, two cells establish a direct, bridge-like connection, called the *pilus*, which brings the cells together. A separate channel is then opened in the respective cell walls, through which a single DNA strand is transferred from the *donor* cell to the *recipient* cell [20]. The importance of this transfer process is that it facilitates the transmission of large, specific genetic messages, which can have arbitrary content. We therefore seek to harness its potential in order to facilitate communication within an engineered cellular population.

In order to achieve this goal, we use *site-specific recombination systems* [22], which allow individual cells to dynamically rewrite their DNA “message”. Recently, three bacterial site-specific recombination systems have been used to implement biocomputing devices. The Cre/*lox* system was used to engineer a genetic switch [23], and the *fim* system was used to engineer both a multiplexer [24] and a sequential switch-based memory [25], [26]. The latter example made use of the Hin/*hix* system, which was also used to solve various small instances of mathematical problems in bacterial populations [27], [28]. These implementations demonstrate the power and applicability of such site-specific recombination systems. Futhermore, as this manuscript was under preparation, Siuti *et al.* [29] published their recombinase-based approach to the implementation of logic and memory functions in *E. coli*, underlining the utility of this approach. We present the results of extensive simulation-based experiments, which support the in-principle feasibility of our approach. This work offers a firm foundation for experimental investigations into distributed multicellular computation using conjugation as a core “technology”.

## Results

We first describe the communication mechanism through conjugation by showing how it may be used, in principle, to implement a single Boolean NOR function. Secondly, we expand the concept by designing a distributed population to implement the exclusive OR (XOR) function, based on mixing three bacterial strains with individual NOR functionality. While using only one site-specific recombination system is sufficient for the NOR-based approach, a combination of two is necessary for the adequate functioning of the XOR computation. For clarity of description, we use abstract labels for the components (except those involved in the recombinase-based logic systems).

### Wiring with conjugative plasmids: 2-strain NOR population

**Figure 1:**
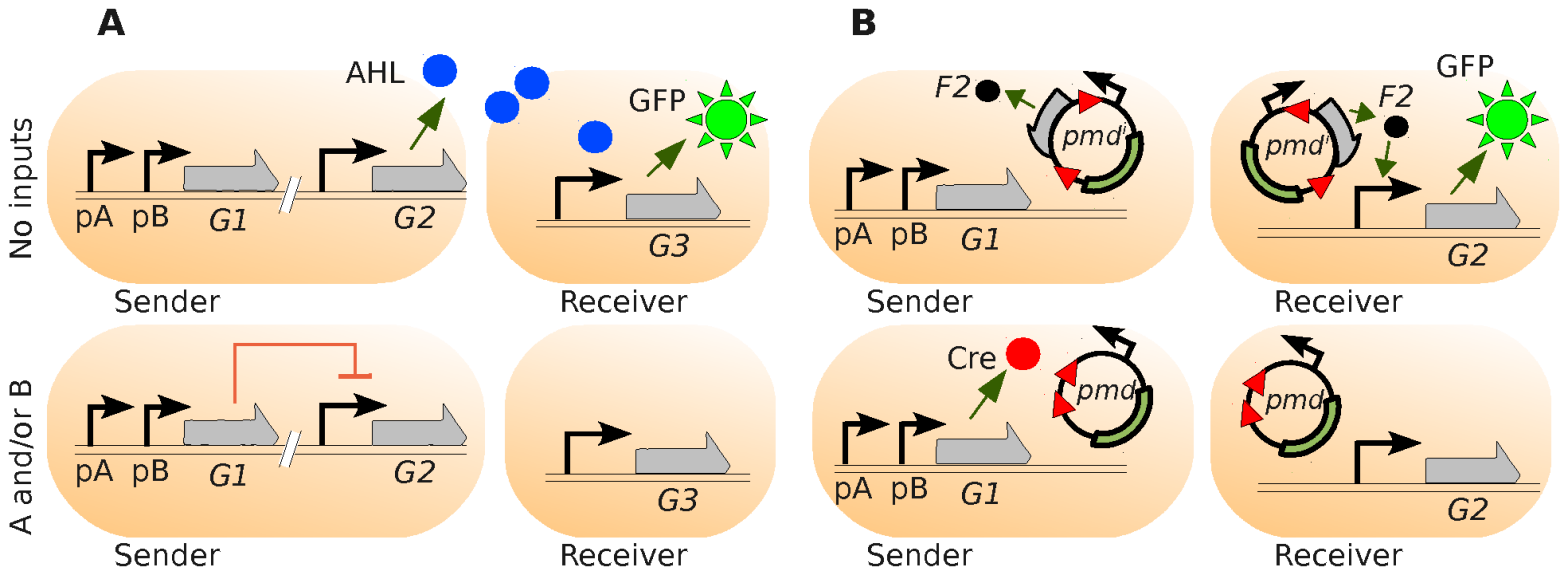
Intercell connection of a NOR logic function. **A**. *Traditional* wiring through AHL signalling. When no inputs (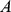 and 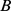) are present (upper row), the AHL quorum-sensing effectors (expressed by gene 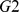 and controlled by a repressible promoter) are produced by the Sender cell and the light (green fluorescent protein, GFP, transcribed by an inducible promoter) is switched on in the Receiver cell. On the other hand, when one or more inputs are present (lower row), AHL production is repressed (via the expression product of gene 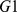) and the fluorescence switched off. **B**. Wiring by using conjugation. In the 0-0 case (no inputs, upper row) the plasmid 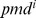 (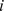 = inducer) *travels* from the Sender to the Receiver cell without modification and induces the expression of GFP. In any other case, the protein Cre is expressed (from gene 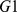) in the Sender, which irreversibly alters the plasmid by deleting the segment between *lox* sites (red triangles). The modified plasmid cannot produce inducers (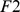), and the Receiver is switched off.

Recall that the two-input NOR function is a negated OR, and thus returns the value 1 if and only if both inputs are zero, and 0 in all other cases. Figure 1A shows how a NOR gate may be constructed using two engineered bacterial strains that communicate via the QS molecule AHL (as in [13]). The evaluation of the NOR function is executed by a *sender* strain, and the output is sent to the *receiver* strain via QS. In this way, QS acts as a *wire* connecting the two components. When no inputs are present (top row of 1A), gene 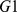 is *off* (not being transcribed), and the AHL signalling molecules (controlled by a repressible promoter) are expressed by 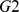. These molecules arrive at the *receiver* cell and, after binding to the corresponding transcription factor, induce expression of gene 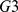 (the reporter gene). This latter product is read as the output of the NOR logic function (fluorescence detection of the amount of green fluorescent protein, GFP). On the other hand, when one or more inputs are present (bottom row, “A and/or B”), their corresponding promoter is activated (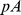 and/or 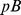), and gene 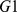 expresses a repressor, which in turns inhibits the production of AHL. Thus, the *receiver* cells are *off*, and no fluorescence is observed.

Our *alternative* design for a distributed NOR gate using conjugation as wiring is shown in Figure 1B. As before, the sender cell computes the NOR function and communicates the result to the receiver cell. The key difference with the previous scheme lies in the communication mechanism; here, we use *conjugative plasmids* [30], rather than QS, to transmit a result. Conjugative plasmids are circular strands of DNA that may be transferred between bacterial cells during the process of conjugation [20], [31]. Although, in the wild, these plasmids allow bacteria to exchange potentially useful genes, here we use them for the transmission of *logical values*.

When no inputs are present (Figure 1B(top row)), promoters 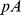 and 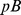 are not induced, and the expression product of 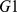 (in this case, protein Cre) is not present in the cell. As a result, a constitutive promoter in the plasmid 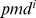 (a plasmid that produces inducers) expresses its downstream gene, which results in inducer 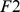.

When the sender cell comes into contact and conjugates with a recipient, thus forming a “wiring” connection, the plasmid is transferred into the receiver cell. Inducers 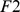 are then produced inside the receiver by plasmid 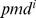, which are in charge of inducing the expression of the reporter gene (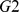). If the resulting product is the green fluorescent protein (GFP), the fluorescence (output) is *turned on*. Otherwise (one or more inputs, bottom row), the protein Cre is expressed, which *deletes* a specific DNA segment surrounded by *lox* sites in the plasmid. As a result, the plasmid 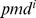 is converted into 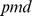 (a plasmid that is unable to produce inducers, due to the lack of the lox-flanked DNA segment). When transferred into the receiver cell, this plasmid will *not* express inducer 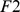, and fluorescence emission will be switched *off*. Thus, the conjugation wire successfully transmits the signal via aplasmid.

It is important to note that when the modified plasmid 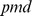 is produced - via deletion - in the sender and transferred to the receiver, it cannot coexist in the latter strain with the previously-introduced unaltered plasmids 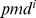 (before the input signals were introduced to the population). Incompatibility is a property of plasmids that contain the same replication genes [32], and is used here to induce “competition”. In this scenario, the population of plasmids in the receiver cells gradually shifts towards the “new” (transmitted) plasmid, as the latter gradually replaces the original. Since both plasmids are viewed as identical from the point of view of replication control, the copies of 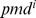 and 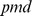 *compete* for an effective resource - the number of plasmids that are allowed in the cell, referred to as the *copy number*. Since copies of 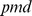 are continuously “pumped” to receiver cells, in practical terms they eliminate 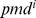. As we see later, this fact has been incorporated into the model, and results in a progressive change in the plasmid population of the receiver, from 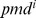 to 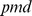.

#### Single cell behaviour

**Figure 2:**
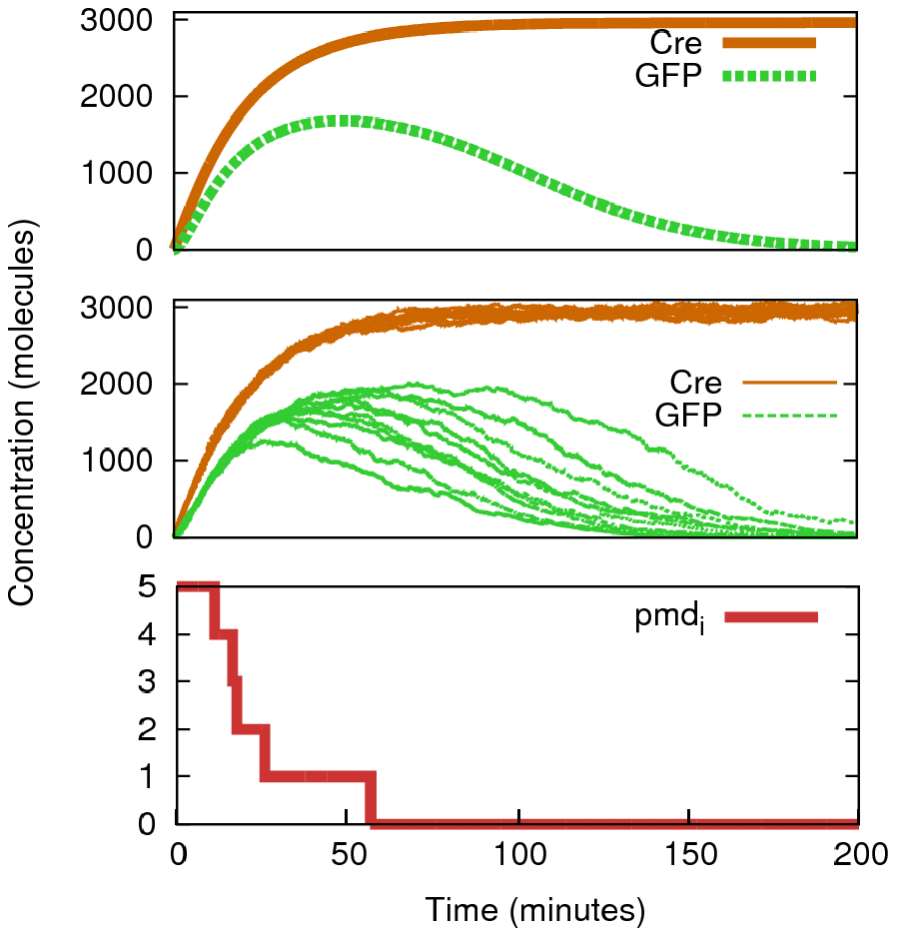
Deterministic and stochastic time evolution of the 1-1 logic case. These results show the simulation of Figure 1B with input molecules (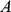 and 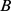) set up to 500 (each) (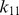 = 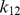 = 500 molecules min^−1^) and the copy number of the plasmid to 5. By monitoring (deterministically, upper graph, one run; stochastically, middle graph, ten runs) proteins Cre (in the Sender) and GFP (in the Receiver) we see how GFP production is initially triggered by those plasmids that have not been modified yet (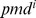). When Cre has been functioning long enough (t
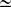
57 min in this simulation) the remaining GFP is only controlled by degradation rates, as no more fluorescent proteins are being expressed. Lower graph shows the stochastic evolution of 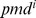 over time, which determines the delay in displaying the correct output according to the NOR logic function (0 output for the 1-1 case).

The simulated single-cell behaviour of this design is observed, over time, in Figure 2, where the logic case 1-1 (both inputs present) is applied to a population initially in the 0-0 state. In this idealised set of simulations, we consider the existence of only one cell of each kind (sender and receiver). We also assume a constant conjugation process (as if the wire was permanently *connected*), so that both cells share the plasmid populations (a theoretical ideal state induced in order to test the individual components of the system). As we observe in both deterministic (top) and stochastic (middle) plots, we see a short *flash* from the receivers (GFP initially being expressed), while the expected output should be “0” (corresponding to the NOR 1-1 case). This is due to the fact that from time 0, the senders transfer plasmid 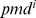 (in its initial configuration) into the receivers. Thus, inducer 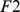 is initially expressed. As Cre is produced, it transforms the plasmid 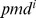 into 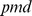 (the transformation of 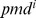 being seen in the bottom graph). When no 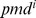 plasmids remain in the ideal system studied in Figure 2, the amount of GFP is controlled only by degradation, and the desired output is reached. Other molecular relations corresponding to the deterministic simulation are shown in Figure S1.

#### Multicellular behaviour

**Figure 3:**
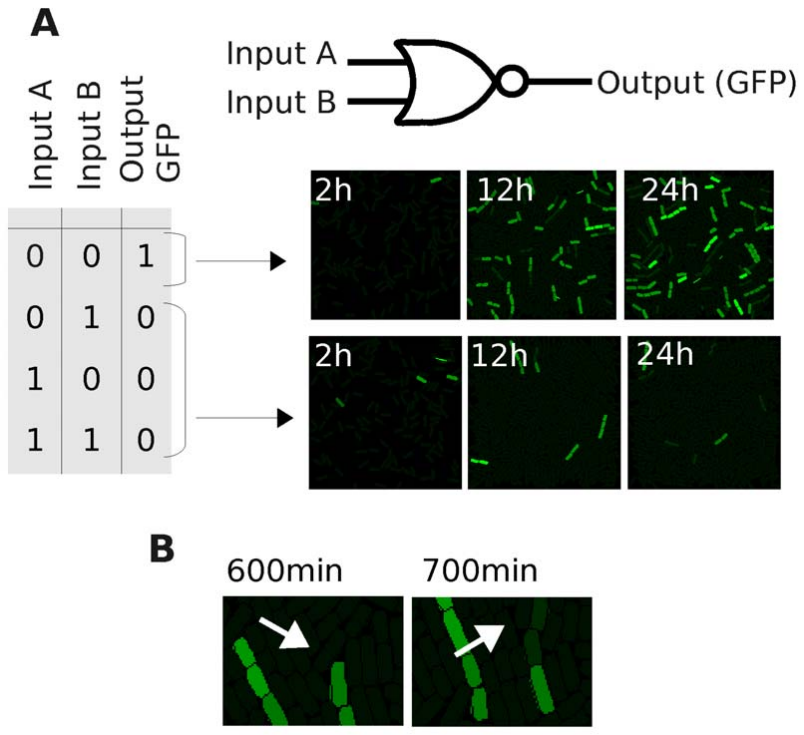
Spatial behaviour of the NOR “wire” over 24 h. **A**. Truth table of the NOR “wire” represented in Figure 1B where inputs A and B can be “0” (0 molecules) or “1” (1000 molecules). Two simulated cell populations are monitored at 2, 12 and 24 h, where the intensity of green colour is directly proportional to the concentration level of GFP (green fluorescent protein). The first one (upper row) corresponds to the logic case 0-0, resulting in an output “1” (lights *on*). The second one (lower row) corresponds to the logic case 1-1 (similar functioning for 0-1 and 1-0 cases) resulting in lights *off*. **B**. Detail of the 1-1 case simulation where the plasmid with inducer (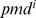) is being replaced by the modified version due to inflow (conjugation) and replication after division. Numbers in cells represent the amount of 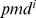 plasmids. After division of a cell with only 1 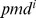, one of the daughter cells will have no copies of this set. Thus, GFP will decay.

A more realistic simulation, which considers physical interactions between cells of a population, is shown in Figure 3. While the cell's logic is simulated via deterministic equations, plasmid numbers (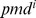 and 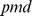) are discrete (see Section Methods for details on discretization). Importantly, deterministic behaviours are not appropriate for low molecular concentrations, and can result in unrealistic behaviours (Figure S2 shows a further study on this issue). Two populations are grown into surfaces with a different distribution of inputs over 24 h. The first (Figure 3A top) has no inputs present and, as a result, recipient cells (receivers) display green light (GFP) corresponding to a logic “1” in output (images from Movie S1). The second population is grown on a simulated surface containing *both* inputs (A and B, Figure 3A) (images from Movie S2). In the latter simulation, we clearly see the effects that the initial *flash* (explained in Figure 2) has on the output of the circuit. While the plasmids 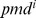 are transformed in the donors at an early stage of the process, those that were initially copied into the receivers are constantly being replicated (until they reach their respective copy number) in the population. This *competition* between 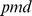 acquisition and 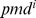 replication (in receivers) results in fluorescent cells after 24 h. However, this fluorescence eventually decays (plasmids 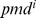 are eventually lost), and we clearly observe the output “0”. Figure 3B shows the process of *losing* plasmid 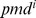 (due to the proliferation of cells without any copy of 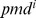). At time 930 min, a fluorescent cell has only one copy of 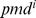, due to the lack of further replication of the existing plasmids in the cell (the rest of the copies are 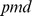). After division (at 950 min) the 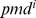 plasmid will go to one of the daughters (all the plasmids are shared randomly). This cell cannot maintain the level of fluorescence after a few min (GFP decay; 990 min). Almost identical behaviour to that seen in input case 1-1 is observed in cases 0-1 and 1-0 (data not shown), as Cre is also expressed in sender cells.

By varying the conjugation frequency we observe changes in the quantitative information of the system, while qualitative behaviours are still the same. Increasing this frequency (Figure S3) causes cells to be overloaded by plasmids (above their copy number), and the replication of plasmids becomes less frequent. For details about cell features, including conjugation frequencies, please see the Methods section.

### Extending the approach: 3-strain XOR population

**Figure 4:**
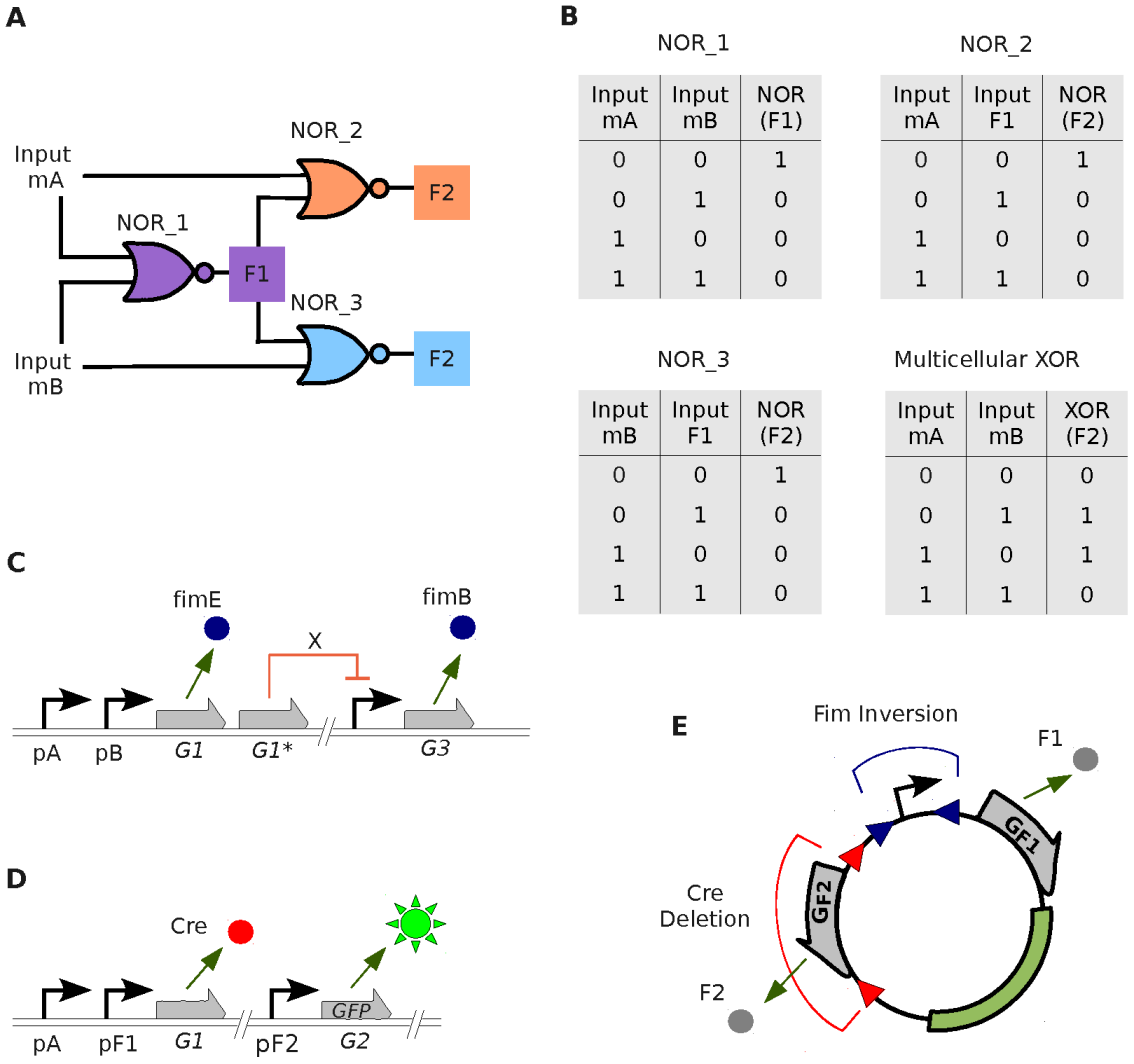
Multicellular design of a distributed XOR circuit. **A**. Schema of the three-strain population and its *connections*. Cell NOR_1 (donor) takes its inputs from the concentration of molecules A and B (inputs of the whole circuit) and its output is named “F1” (which is a specific plasmid configuration). The inputs for cell NOR_2 (recipient) are molecule A and F1. Similarly, the inputs for cell NOR_3 (recipient) are B and F1. The output for the XOR function, “F2” is the combination of outputs from NORs 2 and 3 (green fluorescent protein, GFP, in this example). **B**. Truth tables for each single gate and for the full XOR circuit. **C**. Inside logic of NOR_1. In the case 0-0 (no inputs present), gene 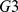 expresses 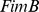 which, in turns, alters the plasmid present in that strain by inverting the promoter in random direction. In any other case, gene 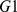 expresses 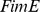 which inverts the promoter region of the plasmid pointing towards gene “F2”; While gene 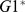 expresses repressor 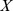 which stops the production of 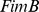. **D**. Inside logic of cell NOR_2. Once the plasmid is in the cell (*wire connected*), if no inputs present, GFP is expressed. Otherwise, 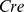 is produced (inducers 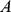 bound to inducible promoter 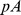 and/or inducer 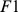 bound to 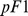) which deletes gene 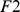 from the plasmid. (Cell NOR_3 has the same circuitry but it is sensitive to input 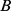 instead of 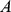). **E**. *Wiring* plasmid. Due to Fim inversion, the promoter can point towards 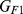 or 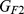. Due to Cre deletion, gene 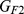 can be removed.

We now show how collections of NOR gates may be connected together in order to compute the exclusive OR (XOR) function, as in [13]. Figure 4 shows the design of a three-strain distributed XOR population, as a proof of principle of the extensibility of the initial approach. The XOR (exclusive OR) function outputs “1” when the inputs are different and “0” when the inputs are the same. Importantly, the inputs are found as *clear* digital values, which are either abundant (“1”) or non-existent (“0”). This avoids potential problems with *half*-values [33]. This logic function may be simulated by connecting three NOR gates, as shown in Figure 4A, which is the configuration of our engineered three-strain population. Exactly the same scheme was used in [13], only with QS molecules connecting the gates. The cells named NOR_1 are the “donors” of the community, and their inputs correspond to the inputs of the whole XOR function (molecules 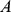 and 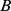). Their output, 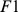 (Figure 4A) encodes the information that travels through the “wire” towards the next logic gates NOR_2 and NOR_3. These latter gates take 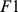 as one of their inputs, and either A or B (respectively) as the other. The output of NOR_2 and NOR_3 cells, which we call 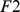 (the same signal), is the final output of the XOR function. In Figure 4B we show the truth tables of individual strains as well as the emergent XOR logic.

The configuration of NOR_1 cells is depicted in Figure 4C. If no inputs are present, gene 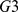 - controlled by a repressible promoter - produces FimB. This causes random inversions of the promoter in the plasmids (Figure 4E). As a result, about half of the plasmids will have the promoter pointing towards the gene named 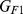, while the rest will have the promoter pointing towards gene 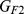. Thus, there will be a positive amount of molecule 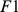 in the environment. This output is taken as a logical “1”. Otherwise, inducible promoters 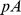 and/or 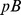 switch *on* genes 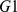 and 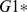, which produce FimE and repressor X respectively. Repressor X inhibits the expression of 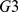. Thus, FimB will disappear from the sender cells, and the presence of FimE causes directional inversion in the promoter, which will then point towards gene 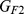. There will thus be a lack of expression products 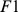 in the environment, which represents a logical “0”. Importantly for our design, as noted in [24], the FimE protein causes directional inversions with nearly 100% fidelity, while FimB causes inversions on both directions equally well [34], [35].

Figure 4D shows the inside *program* of NOR_2 cells. Importantly, this configuration is identical to the combination of the senders and receivers of Figure 1B. If no inputs are present, which means that input molecule 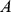 is not in the environment and molecule 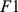 is not being produced by the plasmid (all promoters are pointing at gene 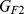), the protein Cre is not expressed. Thus, the segment surrounded by *lox* sites in the plasmid is not deleted. Molecules 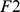 are then effectively expressed and, in turn, induce the production of GFP. If either one or both inputs are present (because 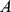 is present and/or 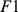 is being expressed by one of the plasmids), protein Cre is expressed. This causes a modification in the plasmid, which loses the 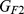 gene region by deletion. No inducers of GFP are present and the fluorescence is *switched off*.

#### Simple behaviour

**Figure 5:**
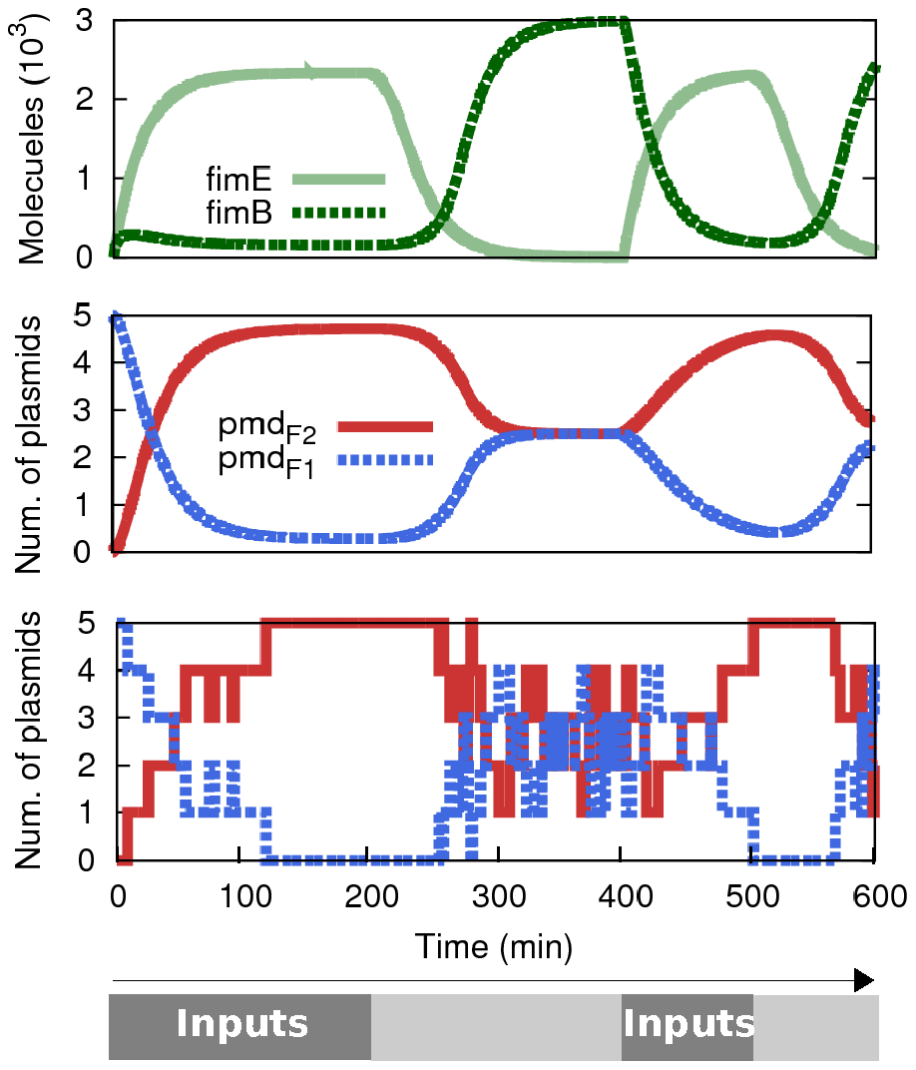
Time evolution of NOR_1 according to a specific input profile. Logic case 1-1 is induced during the intervals [0…200] min and [400…500] min (A and B = 500 molecules -constant entry- during the interval). The case 0-0 domains during the rest of the 600 min. According to that profile we observe the deterministic oscillation of FimE/FimB (top graph) as well as the oscillation between the two possible plasmids in NOR_1 (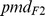 and 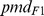). The latter relation is shown deterministically (middle graph) and stochastically (bottom graph) (copy number = 5). Delays in response are due to input degradation times.

A simplified simulation (not considering spatial factors or physical dynamics) of the donor (NOR_1) cells, according to a specific input profile over time, is shown in Figure 5. Input case 1-1 (both inputs present) is induced at 0 min by *introducing* the inputs to the population and maintaining their concentration. At time 200 min we stop introducing inputs, and their concentration is only controlled by degradation rates until case 0-0 is reached. From time 400 min to 500 min we repeat the process. According to that input profile, we observe deterministic oscillation of FimE/FimB (top graph), as well as oscillation between the two possible plasmids in NOR_1 (middle graph) (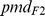, plasmid where the promoter points towards 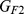 and 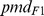, plasmid where the promoter points towards 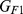). The latter relation is shown stochastically (bottom graph), with a copy number of 5 plasmids, in order to show realistic transitions within discrete numbers. In all graphs we clearly observe the delay produced between the time we stop introducing inputs and the time they effectively disappear from the system. It is important to note that the initial distribution of plasmids (t = 0) is no longer repeated in the simulations. That pristine condition is only valid at the very beginning, and is not representative of the working system. From this simulation we can clearly see how the logic is fully reversible, requiring only changes to inputs, with no modification to the cells. This feature is possible precisely because of the reversible nature of the fimE/B system (i.e., it does not delete the segment).

#### Spatial simulation

**Figure 6:**
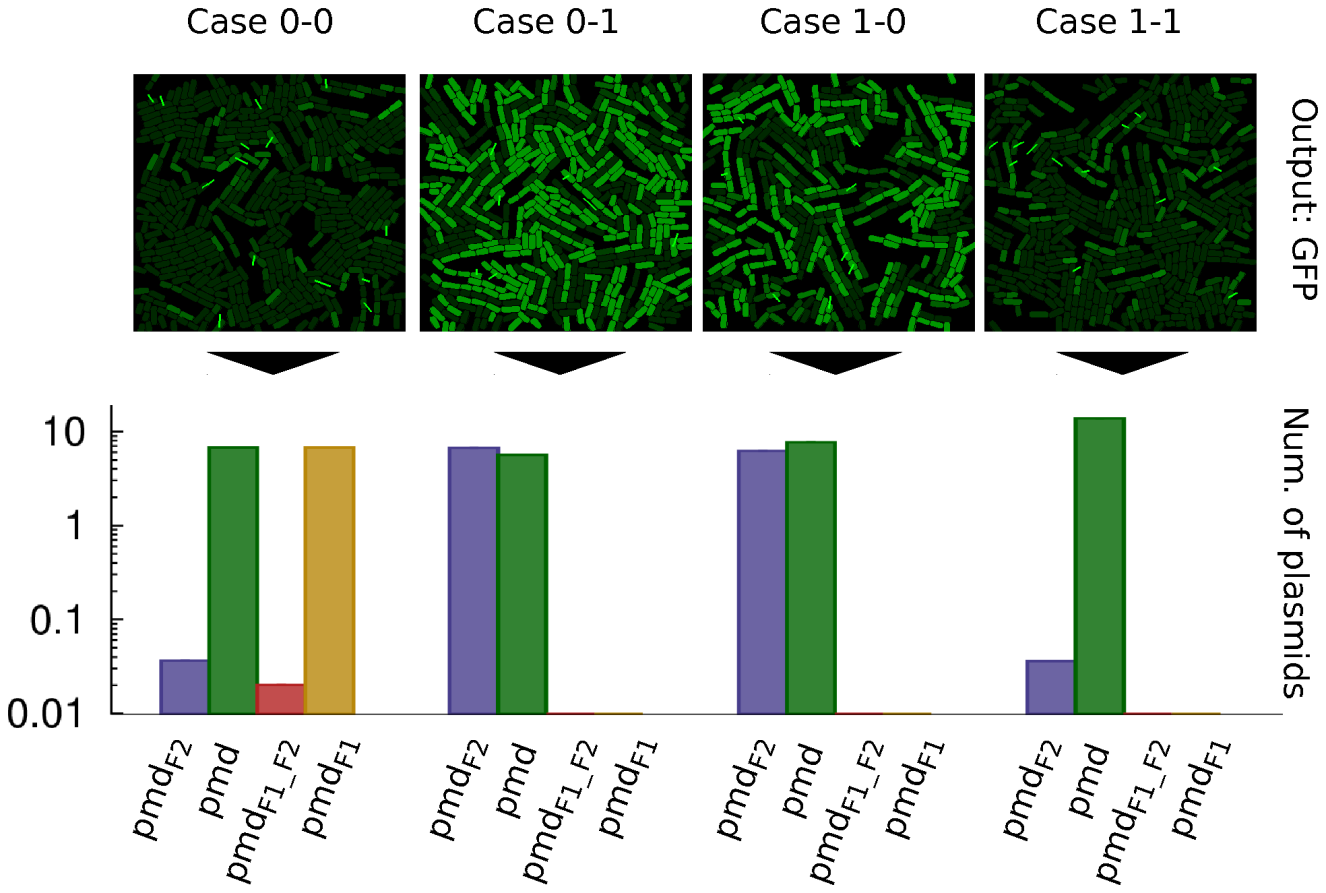
Spatial simulation of the XOR population using the four logic cases. For each input case (0-0, 0-1, 1-0 and 1-1) a snapshot of the population after 32 hours is shown along a profile (bar chart) of the average number of plasmids of each kind (the four possibilities) in recipients (NOR_2 and NOR_3) (copy number = 10). The output, visually identified as green colour cells (from green fluorescent protein, GFP) corresponds to the XOR function. The average number of plasmids 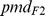 in a population is directly proportional to the fluorescence of it. The “y” axis in the bar graph is displayed in logarithmic scale. Short bright green lines in spatial figures of the population represent a conjugation process currently happening (those “springs” link donor and recipient).

A full spatial simulation of the XOR population is shown in Figure 6. The three different strains are grown over a surface that contains the corresponding combination of inputs (the four logic cases shown). The visual output is represented by the intensity of green colour (high = “1”; low = “0”) proportional to the concentration of GFP in each cell. All snapshots are taken after 32 h *cultivation* (cells have a doubling time of approximately 100 min). As expected, cases 0-1 and 1-0 are the ones that display a positive output following the XOR function. Below each snapshot, we show a graph that pictures the number of plasmids of each four possibilities (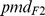, promoter pointing towards 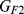; 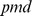, promoter pointing towards 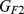 after its deletion; 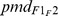, promoter pointing towards 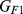; 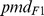, promoter pointing towards 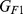 after gene 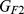 deletion). The number of plasmids is averaged per cell within the receiver population (cells NOR_2 and NOR_3) at the same time as the snapshot is taken (32 h).

The phenomenon of unwanted light *flashes* still occurs at this point in NOR_2 and NOR_3. As described previously, the presence of this phenomenon does not affect the overall functioning of the circuit. Also, conjugation frequency plays an important role in the quantitative behaviour. However, qualitative functioning is still that of an XOR function (Figure S4).

## Discussion

It seems increasingly clear that a significant amount of future research in the field of synthetic biology will be concerned with the construction of engineered microbial consortia [10], [11], [14]. The growing complexity of proposed applications (e.g., in energy, biomedical engineering, or environmental monitoring and bio-remediation [36]) will require approaches that allow a task to be broken down into separate sub-tasks, each of which may be addressed by a separately-optimised population of cells. From an historical perspective, this is entirely consistent with the development of computer software, which has moved from a *monolithic* model (in which an application is self-contained and isolated from other programs) to an object-oriented, *distributed* model, in which a system is viewed as a collection of *interacting entities* [37]. Apart from the benefits accrued from a division of labour, mixed populations are also robust to environmental perturbations, which will be particularly significant in “real world” applications [11]. In order to achieve this distributed, engineered cellular computation, we require a reliable *communication* protocol that will allow cells and populations to exchange information, exert control and influence the overall system behaviour. As has already been demonstrated, several such protocols are possible, based on (for example) quorum sensing (QS) [13], [14] or phage infection [16]. In this paper, we propose a third alternative communication scheme, based on bacterial *conjugation*.

A clear advantage of the proposed system is the *amount* of information that may be communicated. Quorum-sensing systems are based on the exchange of small signalling molecules, which are ideal for global synchronization of a system, where the controlling signal may be relatively simple. However, such systems lack the richness and complexity of cell-cell DNA-based methods (such as conjugation or phage), using which we may exchange relatively large “packets” of genetic material [16]. An added advantage of using conjugation as a wiring system is the requirement for *physical contact* between donor and recipient cells. Thus, if cells are constrained in their movement (e.g., because they are growing on a solid surface), conjugation allows fine-grained *spatial resolution* of computational processes (as opposed to the global “broadcast” model of QS). This will allow, for example, the precise targeting of the *location* of a specific input signal within a monolayer of communicating cells.

Another important benefit of engineered microbial consortia is *modularity* and *reusability* of components [10]. The ability to combine basic components together in order to build larger structures is a fundamental engineering principle, and facilitating the reuse of cellular systems is of paramount importance to practitioners in synthetic biology. We illustrate this with reference to our proposed XOR circuit. Our initial two-strain population implementing the NOR function is effectively non-reusable, as it cannot be “reset”. This problem is solved in the XOR population, where the system may compute indefinitely due to the reversible nature of the fimE/B system.

Computational simulations allow us to “test” proposed cellular systems in many different scenarios. In this paper, we provide not only deterministic/stochastic single-cell simulations (which provide us with an idealised perspective of the system, impossible to achieve *in vivo*), but also multicellular spatial simulations, which allow us to better understand the *population-level* dynamics of the system. Taken together, these computational studies offer a valuable insight into the proposed system, in order to prepare future wet-lab implementations of our design. Such a framework will allow for relatively easy investigation of implementation-specific issues, which may be expensive or time-consuming to test in the laboratory. For example, in future, we may use our *in silico* approach to explore a wider combination of site-specific recombination tools (only two were combined in this work), and how we may harness their different functionalities (such as insertions, cointegrations, deletions and inversions). Implementation of conjugation-based wiring requires the consideration of various practical issues. Most importantly, there are two types of conjugation channels; those that work with bacteria moving freely in a *liquid* medium (liquid maters, e.g., plasmids F and R64), and those that only work on the surface of a *solid* medium (surface maters, e.g., plasmids RP4, R388 and R46) [38]. The latter allow better control of the experiments, since conjugation only starts when the bacterial population is spread on a solid surface (e.g., an agar plate), and conjugation stops when the cells are taken out of the surface [39]. As a result, the experimenter could divide the computing process in steps, if required. Further control over the experimental course may be achieved by selection for any of the computing strains, for instance by applying antibiotic selection. Periodic selection might be necessary since the various strains used in the computation might have different growth rates, particularly if they are loaded with plasmids (cells containing plasmids typically grow more slowly than plasmid-free ones). Therefore, even if the initial concentrations are 50% donors D and 50% recipients R, this ratio can shift rapidly. Growth rates can be adjusted by applying sub-inhibitory concentrations of antibiotics, and the effects of such changes may be checked using our simulations. As stated in the Methods Section, a conjugation experiment can be prolonged by mixing the cells of a mating and plating out again on fresh plates. Additional donors (or recipients) may be added at this stage to equilibrate the D/R ratio.

Another advantage of our system, which provides an obvious line of enquiry for future work, is the potential *scalability* of the system. There are many plasmid conjugation systems that work in an essentially orthogonal manner (meaning each plasmid uses its own DNA transport system, and the different systems do not interfere, even if acting on the same cell). This is easily understood, since each conjugative system promotes only the transfer of the plasmid it recognizes as its own [20]. In the *E. coli* bacterium there are at least 12 different conjugative systems, coded by compatible plasmids, that can act *together* in the *same* cell [40]. Many strains of *E. coli* isolated from clinical settings carry three or more plasmids, and these assemblies are stable and evolutionarilyy successful [41]. Moreover, a given conjugative plasmid can bring about the mobilization of several independent mobilizable plasmids [42]. Thus, the numbers of potential orthogonal systems quickly escalate.

We fully expect future work in synthetic biology to adhere closely to this model, in which computational simulations and laboratory investigations are inextricably linked in a tight cycle of feedback. Our proposed system offers one possible addition to the ever-growing “toolbox” available to biological engineers, and we hope that experimental validation of its basic principles will be quickly forthcoming.

## Methods

### Modelling genetic logic

The model used for all simulations is supplied in this Section. We make the assumption that modelling the translation processes and the slow transcriptions (basal rates) make no qualitative difference. Thus, we adopt the following two simplifications in our system: 1) transcription and translation are joined into one single process called “transcription”, and 2) basal transcription rates are considered to be null in the system.

Ordinary differential equations (ODEs) from 1 to 13 explain the deterministic dynamics of the first approach (2-strain NOR population) using an *idealized* two-cell environment where physical contact is constant:
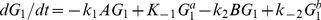
(1)

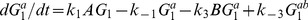
(2)

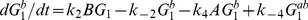
(3)

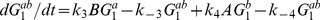
(4)

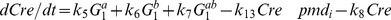
(5)

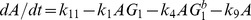
(6)

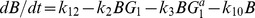
(7)

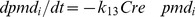
(8)

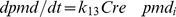
(9)

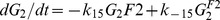
(10)

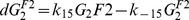
(11)

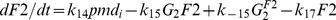
(12)

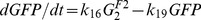
(13)where 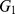 denotes the gene of the sender cell without inputs bound; 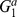, 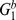 and 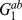 denotes the same gene with inputs A, B and both bound (respectively) to its promoters; 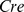 is the Cre protein; 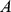 and 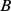 are the inputs; 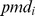 and 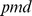 the plasmid with the segment that contains the gene in charge of expressing inducers or without it (respectively); 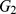 denotes the gene in the receiver cell without inducer bound; 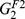 is the same gene with inducers bound to its promoter; 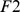 are the inducers expressed by the *wiring* plasmid; and 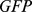 are the green fluorescent proteins.

Biochemical reactions and kinetic rate values (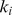) are detailed in Text S1. All rates are specified within standard ranges found in the literature [43]–[48]. Kinetic rate *k13* has been fixed according to the experimental results of [23]. Importantly, similar parameters have the similar values, following the objective of defining as general a system as possible (free from parameter constraints that could compromise future validation). A perturbation analysis is performed (Figure S5) by adding Gaussian noise to every rate (being the mean the original value and the standard deviation 20% of the mean) and running the simulations several times. This analysis reinforces our conclusion about the qualitative robustness of our model. The quantitative behaviour may change without altering the overall goal of the computational process.

Stochastic simulations, that take into account the randomness of the chemical reactions, used the Gillespie algorithm [49] (using reactions described in Text S1). In this approach, time is discrete and the rates (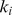) indicate the propensity of each reaction to happen.

The deterministic behaviour of cells NOR_1 (component of the 3-strain XOR population) are described by equations 14–20:
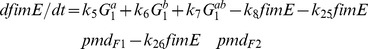
(14)

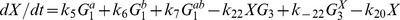
(15)

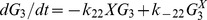
(16)

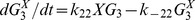
(17)

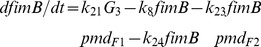
(18)

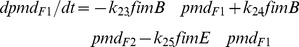
(19)

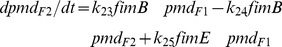
(20)where 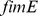 and 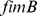 denote the inversion proteins; 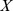 represents the repressor; 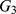 is the gene that expresses 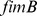; 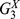 denotes the same gene when the repressor 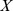 is bound to its promoter; and 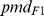 and 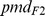 are the possible plasmid configurations inside NOR_1 cells. Equations 1, 2, 3, 4, 6 and 7 are also used in this example as they define dynamics for existing components in NOR_1 cells. Reactions and rates are, as before, detailed in Text S1. Importantly, 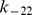 is a low value in order to simulate a strong repression done by 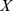.

Separately, NOR_2 cells are simulated by ODEs 21 to 24. As stated in Section Results, the inside *program* of these cells are similar to the combination of both senders and receivers of the 2-strain NOR design (Figure S6 shows the transition to logic case 1-0 inside a NOR_2 cell). The only difference relies on the four plasmid configurations (instead of two) that can be present in the cell. Thus, their equations are the same as in the NOR example but with some modifications:
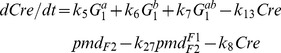
(21)

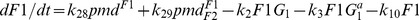
(22)

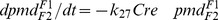
(23)

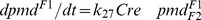
(24)where 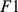 is the expression product that induces the production of Cre; 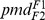 and 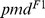 are the plasmid with or without (respectively) gene 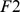 when the promoter is pointing towards gene F1. The plasmids 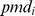 and 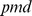 (equations 8 and 9 also used in cells NOR_2) make reference to plasmids 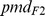 and 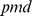. The gene 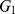 inside the NOR_1 cell is not the same than the gene 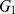 inside the NOR_2 cell, altough they share the label. Stochastic simulations are done (via Gillespie algorithm) by using the biochemical reactions with the kinetic rates shown in Text S1.

The code of the previous model is included in Code S1 (all code in Python). This code is enough to reproduce Figures 2, 5, S1, S2 and S6. The spatial figures (Figures 3, 6, S3 and S4) make use of the package DiSCUS as explained in the following section.

### Population dynamics

A simulation tool for population dynamics is needed in order to get realistic spatial results (Figures 3, 6, S3 and S4) for the whole system behaviour. We present a simulation framework for bacterial growth, movement and horizontal gene transfer called DiSCUS (DIscrete Simulation of Conjugation Using Springs - http://code.google.com/p/discus/). Using an agent-based model (AbM) approach, our software combines the management of intercellular interactions with the simulation of intracellular genetic networks. AbMs are widely used to study microbial growth and biofilm formation [50]–[53] and conjugation has also been included in several simulations [31], [54], [55], but these (unrealistically) consider cells as abstract circular objects. Having rod-shaped cells (as in [56]–[59]) is essential to obtain accurate conjugation dynamics. Thus, DiSCUS is the first platform, to our knowledge, to offer the possibility of simulating conjugation dynamics in rod-shape bacteria.

ODEs are introduced *inside* every cell independently, so each bacterium runs its own copy of the circuit according to its design. While the circuit is simulated deterministically, the plasmid number is always discretized using several build-in functions. The discrete number corresponding to the plasmid concentration is used when: a) the cell divides, when a random selection of the plasmids will be part of each daughter (without losing plasmids); b) the plasmids have lower copy number than the limit, in which case they increase their copies; and c) the cell transfers (through conjugation) a small number of plasmids (1–3) to the recipient. The discretized values are always used to update deterministic concentrations. This process is essential to assure the numbers are kept consistent and realistic.

Conjugation events take place while the cells carry out their normal growing activity. DiSCUS handles conjugation processes through a probability distribution that can be easily tuned to correspond to the behaviour of different cell types. The *low* and *high* conjugation frequencies used in this paper are obtained via visual validation against real data taken from a recent study [60] (Figure S7A–C) where a difference is established between original donors (*low* conjugation frequency) and new donors or *transconjugants* (*high* conjugation probability). Biomechanical validation of DiSCUS (Figure S7D–I) against real data [59] has also been performed. In our design there is only one conjugation frequency, as transconjugant cells are unable to conjugate between them or back to donors (due to the fact that they do not carry a complete genetic transfer system). However, we run duplicate simulations considering both alternative frequencies (*low*: Figures 3 and S4; *high*: Figures 6 and S3) in order to explore the range of correct functioning of the system. We observe quantitative changes, but not significant alterations in the qualitative logic behaviour of the circuits. In order to allow conjugation to achieve complete infection of the recipient population during the experiment, the cells were mixed (shuffled) every 400 min so that new pairs donor-receiver can arise in the population (this phenomenon has been tested and validated experimentally in [39]; we provide simulated proof in Figure S8).

## Supporting Information

Code S1 **Python code for the intra-cell circuits.** Equations 1 to 13, that explain the deterministic behaviour of the first 2-strain NOR population are coded in the file *NORdet.py* while the rates are simulated stochastically (Gillespie) in the file *NOTsto.py*. Regarding the XOR example, the NOR_1 strain is simulated deterministically in *XOR(NOR1)det.py* (ODEs 14–20) and stochastically in *XOR(NOR1)sto.py*; The ODEs for the NOR_2 strain are coded in *XOR(NOR2)det.py*.
(ZIP)

Figure S1
**Molecular deterministic relations in the simulation of** **Figure 1B** **.** Using the same simulation of Figure 2 (case 1-1), these graphs show the behavioural changes in the relation between different molecules when the rate 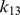 (Cre deletion rate) is changed. All graphs show information at time = 200 min. **A**. Cre proteins and altered plasmids 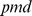 (copy number = 1). **B**. Cre proteins and GFP molecules. **C**. F2 inducers and GFP molecules.(EPS)

Figure S2
**Comparison of stochastic and deterministic behaviour in plasmid concentration.** Twelve different simulations of Figure 1B (case 1-1) over 150 min. Only 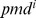 and 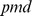 are monitored. Copy number equals 1 (six graphs on the left) and 20 (six graphs on the right). Rate 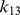 equals 2E-5 (top row), 2E-6 (middle row) and 2E-3 (bottom row). The unrealistic transitions of deterministic simulations (there is no such a thing as “0.243” plasmids in a cell) match better the stochastic transitions with bigger copy number. Also, big values of 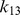 will give better deterministic approximations (due to small delay in conversion).(EPS)

Figure S3 **Alternative spatial simulations to** **Figure 3** **with greater conjugation frequency.** Red outline cells: donors (senders). Blue outline cells: recipients (receivers). Green colour intensity proportional to GFP concentration level (output). **A**. Logic 0-0 case. **B**. Logic 1-1 case. Bars in bar graphs proportional to plasmid numbers in the whole population (excluding sender cells).
(EPS)

Figure S4
**Alternative spatial simulation for case 0-0 in** **Figure 6** **with lower conjugation frequency.** Snapshot of an XOR population after 32 h when no inputs are present (right) and bar graph showing the average number of plasmids (per configuration) in receivers (left) at the same instant (*y* axis in logarithmic scale). By setting up the conjugation frequency to a low value, the number of plasmids 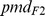 (which control the output) increases. As a result, some cells display a positive output (GFP) when they should not according to the XOR function. However, this positive output is residual.(EPS)

Figure S5
**Perturbation analysis of model reaction rates.** In each graph (A–D), the results of 50 simulations are shown, 45 of which use a perturbed set of reaction rates, and 5 of which use the original rates (control set, above dashed horizontal lines in graphs). In each experiment, we perturb every rate by adding Gaussian noise to the original value (stardard deviation is 20%). A,B: Perturbation analysis of the model (Figure 2) for the 2-strain NOR population (23 rates), where the graphs show the effect of the output (GFP) when the inputs are 0-0 and 1-1 respectively. C,D: perturbation analysis for the XOR model (Figure 5), NOR_1-strain (22 rates; inputs considered constrant in the profile), where the graphs measure the level of the fimE protein and the 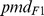 plasmids, respectively.(EPS)

Figure S6
**Simulation results of NOR_2 during its logic 1-0 case.** This case means that molecules 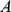 are present (logic “1” = 1000 molecules at the start; 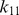 = 500 molecules min^−1^) and the plasmids coming from donors (NOR_1) are all in configuration 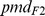. In this scenario, we would expect a logic “0” in the output (no GFP). However, we can observe an initial GFP expression (bottom graph) which corresponds to the time delay needed to change the configuration of the plasmid from 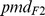 to 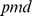 (top graph). After GFP is no longer expressed, its degradation controls the concentration. Copy number = 5.(EPS)

Figure S7 **Validation of DiSCUS.** Left (figures A–C): validation of conjugation dynamics using real data. **A**. Figure extracted from [60] where a colony of *Pseudomonas putida* is divided into dark red donor cells (DsRed), yellow recipient cells (YFP) and transconjugants, expressing both yellow and green light (YFP and GFP). The upper row shows the transconjugant signal, and the bottom row shows the whole community. **B and C**. Simulation results. Two simulations of similar colonies are recorded over exactly the same time intervals (min). The colours of the cells match the colours observed in **A**. Right (graphs D–I): validation of cell movement using real data. Graphs **D**, **F** and **H** are extracted from [59], and show experimental results of *Escherichia coli* growth regarding density, velocity gradient and ordering (respectively). Graphs **E**, **G** and **I** correspond to simulations in similar conditions to [59], for the same parameters (density, velocity gradient and ordering respectively). Test 1, 2 and 3 in graphs correspond to different spatial distribution of cels inside the microfluidic chanel.
(EPS)

Figure S8 **Effects of manual mixing on conjugation frequency.** **A**. Recipient-trapping behaviour ofa population wth donors (red), transconjugants (green) and recipients (yellow). Two snapshots depict clearly-observed clusters. **B**. Population arfter random mixing, where the clusters are automatically dissolved. **C**. Graph showing conjugation frequencies (Y = T/(R+T)) of 560-minute experiments (ratio D/R = 50%). Blue bars represent Y on an untouched population, while red bars represent Y wen the population is mixed at 420 min. The two sets of bars corespond to experiments with different cell dimensions (1×3 -left- and 1×2 -right-). Error bars show variation across 15 experiments of each class.
(EPS)

Movie S1 **The 2-strain NOR population (0-0).** Spatial simulation of the 0-0 logic case from where images in Figure 3 are taken. 24-hour video where the community is shuffled randomly every 400 min as stated in the text.
(MOV)

Movie S2 **The 2-strain NOR population (1-1).** Spatial simulation of the 1-1 logic case from where images in Figure 3 are taken. 24-hour video where the community is shuffled randomly every 400 min as stated in the text.
(MOV)

Text S1 **Reactions and rates used in the simulations.** This text contains the chemical reactions and the rate values used in all simulations of the paper.
(PDF)

## References

[1] Oldham P, Hall S, Burton G (2012) Synthetic biology: mapping the scientific landscape. PLoS One 7: e34368. 22539946 10.1371/journal.pone.0034368 PMC3335118

[2] Heinemann M, Panke S (2006) Synthetic biology{putting engineering into biology. Bioinformatics 22: 2790–9. 16954140 10.1093/bioinformatics/btl469

[3] Andrianantoandro E, Basu S, Karig DK, Weiss R (2006) Synthetic biology: new engineering rules for an emerging discipline. Mol Syst Biol 2: 2006.0028. 10.1038/msb4100073 PMC1681505 16738572

[4] De Lorenzo V, Danchin A (2008) Synthetic biology: discovering new worlds and new words. EMBO reports 9: 822–827. 18724274 10.1038/embor.2008.159 PMC2529360

[5] Elowitz MB, Leibler S (2000) A synthetic oscillatory network of transcriptional regulators. Nature 403: 335–338. 10659856 10.1038/35002125

[6] Gardner TS, Cantor CR, Collins JJ (2000) Construction of a genetic toggle switch in Escherichia coli. Nature 403: 339–342. 10659857 10.1038/35002131

[7] Lou C, Liu X, Ni M, Huang Y, Huang Q, et al (2010) Synthesizing a novel genetic sequential logic circuit: a push-on push-off switch. Molecular systems biology 6. 10.1038/msb.2010.2 PMC2858441 20212522

[8] Purcell O, Savery NJ, Grierson CS, di Bernardo M (2010) A comparative analysis of synthetic genetic oscillators. J R Soc Interface 7: 1503–24. 20591848 10.1098/rsif.2010.0183 PMC2988261

[9] Purcell O, di Bernardo M, Grierson CS, Savery NJ (2011) A multi-functional synthetic gene network: a frequency multiplier, oscillator and switch. PLoS One 6: e16140. 21359152 10.1371/journal.pone.0016140 PMC3040778

[10] Macía J, Posas F, Solé RV (2012) Distributed computation: the new wave of synthetic biology devices. Trends Biotechnol 30: 342–9. 22516742 10.1016/j.tibtech.2012.03.006

[11] Brenner K, You L, Arnold FH (2008) Engineering microbial consortia: a new frontier in synthetic biology. Trends Biotechnol 26: 483–9. 18675483 10.1016/j.tibtech.2008.05.004

[12] Basu S, Gerchman Y, Collins CH, Arnold FH, Weiss R (2005) A synthetic multicellular system for programmed pattern formation. Nature 434: 1130–4. 15858574 10.1038/nature03461

[13] Tamsir A, Tabor JJ, Voigt Ca (2011) Robust multicellular computing using genetically encoded NOR gates and chemical ‘wires’. Nature 469: 212–5. 21150903 10.1038/nature09565 PMC3904220

[14] Regot S, Macia J, Conde N, Furukawa K, Kjellen J, et al (2011) Distributed biological computation with multicellular engineered networks. Nature 469: 207–211. 21150900 10.1038/nature09679

[15] Goñi-Moreno A, Redondo-Nieto M, Arroyo F, Castellanos J (2011) Biocircuit design through engineering bacterial logic gates. Natural Computing 10: 119–127.

[16] Ortiz M, Endy D (2012) Engineered cell-cell communication via dna messaging. Journal of Biological Engineering 6: 16. 22958599 10.1186/1754-1611-6-16 PMC3509006

[17] Atkinson S, Williams P (2009) Quorum sensing and social networking in the microbial world. J R Soc Interface 6: 959–78. 19674996 10.1098/rsif.2009.0203 PMC2827448

[18] Danino T, Mondragón-Palomino O, Tsimring L, Hasty J (2011) A synchronized quorum of genetic clocks. Nature 463: 326–330. 10.1038/nature08753 PMC2838179 20090747

[19] Tatum EL, Lederberg J (1947) Gene recombination in the bacterium escherichia coli. Journal of Bacteriology 53: 673–684. 16561324 10.1128/jb.53.6.673-684.1947 PMC518375

[20] De La Cruz F, Frost LS, Meyer RJ, Zechner EL (2010) Conjugative dna metabolism in gramnegative bacteria. FEMS Microbiology Reviews 34: 18–40. 19919603 10.1111/j.1574-6976.2009.00195.x

[21] Llosa M, Gomis-Rth FX, Coll M, Cruz Fdl (2002) Bacterial conjugation: a two-step mechanism for dna transport. Molecular Microbiology 45: 1–8. 12100543 10.1046/j.1365-2958.2002.03014.x

[22] Grindley ND, Whiteson KL, Rice PA (2006) Mechanisms of site-specific recombination*. Annual Review of Biochemistry 75: 567–605. 10.1146/annurev.biochem.73.011303.073908 16756503

[23] Yamanishi M, Matsuyama T (2012) A modified cre-lox genetic switch to dynamically control metabolic ow in saccharomyces cerevisiae. ACS Synthetic Biology 1: 172–180. 23651155 10.1021/sb200017p

[24] Moon TS, Clarke EJ, Groban ES, Tamsir A, Clark RM, et al (2011) Construction of a genetic multiplexer to toggle between chemosensory pathways in escherichia coli. Journal of Molecular Biology 406: 215–227. 21185306 10.1016/j.jmb.2010.12.019 PMC3033806

[25] Ham TS, Lee SK, Keasling JD, Arkin AP (2008) Design and construction of a double inversion recombination switch for heritable sequential genetic memory. PLoS ONE 3: e2815. 18665232 10.1371/journal.pone.0002815 PMC2481393

[26] Ham TS, Lee SK, Keasling JD, Arkin AP (2006) A tightly regulated inducible expression system utilizing the fim inversion recombination switch. Biotechnology and Bioengineering 94: 1–4. 16534780 10.1002/bit.20916

[27] Haynes K, Broderick M, Brown A, Butner T, Dickson J, et al (2008) Engineering bacteria to solve the burnt pancake problem. Journal of Biological Engineering 2: 8. 18492232 10.1186/1754-1611-2-8 PMC2427008

[28] Baumgardner J, Acker K, Adefuye O, Crowley S, DeLoache W, et al (2009) Solving a Hamiltonian path problem with a bacterial computer. Journal of Biological Engineering 3: 11. 19630940 10.1186/1754-1611-3-11 PMC2723075

[29] Siuti P, Yazbek J, Lu TK (2013) Synthetic circuits integrating logic and memory in living cells. Nature Biotechnology 10.1038/nbt.2510 23396014

[30] Garcillán-Barcia MP, de la Cruz F (2008) Why is entry exclusion an essential feature of conjugative plasmids? Plasmid 60: 1–18. 18440635 10.1016/j.plasmid.2008.03.002

[31] Gregory R, Saunders JR, Saunders VA (2008) Rule-based modelling of conjugative plasmid transfer and incompatibility. Biosystems 91: 201–15. 18023962 10.1016/j.biosystems.2007.09.003

[32] Novick RP (1987) Plasmid incompatibility. Microbiological Reviews 51: 381–395. 3325793 10.1128/mr.51.4.381-395.1987 PMC373122

[33] Goñi-Moreno A, Amos M (2012) A reconfigurable nand/nor genetic logic gate. BMC Systems Biology 6: 126. 22989145 10.1186/1752-0509-6-126 PMC3776446

[34] Gally DL, Bogan JA, Eisenstein BI, Blomfield IC (1993) Environmental regulation of the fim switch controlling type 1 fimbrial phase variation in escherichia coli k-12: effects of temperature and media. Journal of Bacteriology 175: 6186–6193. 8104927 10.1128/jb.175.19.6186-6193.1993 PMC206713

[35] McClain MS, Blomfield IC, Eisenstein BI (1991) Roles of fimb and fime in site-specific dna inversion associated with phase variation of type 1 fimbriae in escherichia coli. Journal of Bacteriology 173: 5308–5314. 1679430 10.1128/jb.173.17.5308-5314.1991 PMC208240

[36] Khalil AS, Collins JJ (2010) Synthetic biology: applications come of age. Nature Reviews Genetics 11: 367–379. 10.1038/nrg2775 PMC2896386 20395970

[37] Meyer B (1988) Object-Oriented Software Construction, volume 2. Prentice Hall, New York.

[38] Smillie C, Garcilln-Barcia MP, Francia MV, Rocha EPC, de la Cruz F (2010) Mobility of plasmids. Microbiology and Molecular Biology Reviews 74: 434–452. 20805406 10.1128/MMBR.00020-10 PMC2937521

[39] del Campo I, Ruiz R, Cuevas A, Revilla C, Vielva L, et al (2012) Determination of conjugation rates on solid surfaces. Plasmid 67: 174–182. 22289895 10.1016/j.plasmid.2012.01.008

[40] Garcilln-Barcia MP, Francia MV, De La Cruz F (2009) The diversity of conjugative relaxases and its application in plasmid classification. FEMS Microbiology Reviews 33: 657–687. 19396961 10.1111/j.1574-6976.2009.00168.x

[41] Grad YH, Godfrey P, Cerquiera GC, Mariani-Kurkdjian P, Gouali M, et al (2013) Comparative genomics of recent shiga toxin-producing escherichia coli o104:h4: Short-term evolution of an emerging pathogen. mBio 4. 10.1128/mBio.00452-12 PMC3551546 23341549

[42] Cabezn E, Sastre J, de la Cruz F (1997) Genetic evidence of a coupling role for the trag protein family in bacterial conjugation. Mol Gen Genet 254: 400–6. 9180693 10.1007/s004380050432

[43] Dublanche Y, Michalodimitrakis K, Kummerer N, Foglierini M, Serrano L (2006) Noise in transcription negative feedback loops: simulation and experimental analysis. Molecular systems biology 2. 10.1038/msb4100081 PMC1681513 16883354

[44] Goñi-Moreno A, Amos M (2012) Continuous computation in engineered gene circuits. Biosystems 109: 52–56. 22387968 10.1016/j.biosystems.2012.02.001

[45] Balagaddé FK, Song H, Ozaki J, Collins CH, Barnet M, et al (2008) A synthetic Escherichia coli predator-prey ecosystem. Mol Syst Biol 4: 187. 18414488 10.1038/msb.2008.24 PMC2387235

[46] Andersen JB, Sternberg C, Poulsen LK, Bjorn SP, Givskov M, et al (1998) New unstable variants of green uorescent protein for studies of transient gene expression in bacteria. Appl Environ Microbiol 6: 2240–2246. 10.1128/aem.64.6.2240-2246.1998 PMC106306 9603842

[47] Bolouri H, Davidson EH (2003) Transcriptional regulatory cascades in development: Initial rates, not steady state, determine network kinetics. Proceedings of the National Academy of Sciences 100: 9371–9376. 10.1073/pnas.1533293100 PMC170925 12883007

[48] de Leon SBT, Davidson EH (2009) Modeling the dynamics of transcriptional gene regulatory networks for animal development. Developmental Biology 325: 317–328. 19028486 10.1016/j.ydbio.2008.10.043 PMC4100934

[49] Gillespie DT (1977) Exact stochastic simulation of coupled chemical reactions. The Journal of Physical Chemistry 81: 2340–2361.

[50] Gorochowski TE, Matyjaszkiewicz A, Todd T, Oak N, Kowalska K, et al (2012) Bsim: an agentbased tool for modeling bacterial populations in systems and synthetic biology. PLoS One 7: e42790. 22936991 10.1371/journal.pone.0042790 PMC3427305

[51] Kreft JU, Booth G, Wimpenny JWT (1998) Bacsim, a simulator for individual-based modelling of bacterial colony growth. Microbiology 144: 3275–3287. 9884219 10.1099/00221287-144-12-3275

[52] Xavier JB, Picioreanu C, van Loosdrecht MCM (2005) A framework for multidimensional modeling of activity and structure of multispecies biofilms. Environ Microbiol 7: 1085–103. 16011747 10.1111/j.1462-2920.2005.00787.x

[53] Lardon LA, Merkey BV, Martins S, Dotsch A, Picioreanu C, et al (2011) idynomics: nextgeneration individual-based modelling of biofilms. Environ Microbiol 13: 2416–34. 21410622 10.1111/j.1462-2920.2011.02414.x

[54] Krone SM, Lu R, Fox R, Suzuki H, Top EM (2007) Modelling the spatial dynamics of plasmid transfer and persistence. Microbiology 153: 2803–16. 17660444 10.1099/mic.0.2006/004531-0 PMC2613009

[55] Merkey BV, Lardon LA, Seoane JM, Kreft JUU, Smets BF (2011) Growth dependence of conjugation explains limited plasmid invasion in biofilms: an individual-based modelling study. Environ Microbiol 13: 2435–52. 21906217 10.1111/j.1462-2920.2011.02535.x

[56] Melke P, Sahlin P, Levchenko A, Jönsson H (2010) A cell-based model for quorum sensing in heterogeneous bacterial colonies. PLoS Comput Biol 6: e1000819. 20585545 10.1371/journal.pcbi.1000819 PMC2887461

[57] Rudge TJ, Steiner PJ, Phillips A, Haseloff J (2012) Computational modeling of synthetic microbial biofilms. ACS Synthetic Biology 1: 345–352. 23651288 10.1021/sb300031n

[58] Cho H, Jönsson H, Campbell K, Melke P, Williams JW, et al (2007) Self-organization in highdensity bacterial colonies: efficient crowd control. PLoS Biol 5: e302. 18044986 10.1371/journal.pbio.0050302 PMC2043048

[59] Volfson D, Cookson S, Hasty J, Tsimring LS (2008) Biomechanical ordering of dense cell populations. Proceedings of the National Academy of Sciences 105: 15346–15351. 10.1073/pnas.0706805105 PMC2563119 18832176

[60] Seoane J, Yankelevich T, Dechesne A, Merkey B, Sternberg C, et al (2011) An individual-based approach to explain plasmid invasion in bacterial populations. FEMS Microbiology Ecology 75: 17–27. 21091520 10.1111/j.1574-6941.2010.00994.x

