# Supplementary material for: Multicellular Computing Using Conjugation for Wiring
Source: PLoS One. 2013 Jun 20;8(6):e65986. doi: 10.1371/journal.pone.0065986 (PMC3688716; doi:10.1371/journal.pone.0065986)
Supplement: Text S1 — Reactions and rates used in the simulations. This text contains the chemical reactions and the rate values used in all simulations of the paper. (PDF) [file pone.0065986.s012.pdf]

Reactions & Rates

Multicellular computing using conjugation for wiring

In this document we describe the reactions and rates used for all simulations used in the paper. From these reactions, we derive the ODEs (Ordinary Differential Equations) shown in the main text. Important note: due to the big number of reactions, the processes of transcription and translation are joined into a single process (for simplicity we call this process *transcription* in this document).

First approach: single "wire"

The set of reactions that describes the behaviour of the circuit depicted in Figure 1B are represented below (rates from 1 to 18):

$$Activation/deactivation: \quad A + G_1 \underset{k_{-1}}{\overset{k_1}{\rightleftharpoons}} G_1^a \tag{1}$$

$$Activation/deactivation: \quad B + G_1 \underset{k_{-2}}{\overset{k_2}{\rightleftharpoons}} G_1^b \tag{2}$$

$$Upgrade/downgrade\ transcription: \quad B + G_1^a \underset{k_{-3}}{\overset{k_3}{\rightleftharpoons}} G_1^{ab} \tag{3}$$

$$Upgrade/downgrade\ transcription: \quad A + G_1^b \underset{k_{-4}}{\overset{k_4}{\rightleftharpoons}} G_1^{ab} \tag{4}$$

$$Transcription: \quad G_1^a \xrightarrow{k5} G_1^a + Cre \tag{5}$$

$$Transcription: \quad G_1^b \xrightarrow{k6} G_1^b + Cre \tag{6}$$

$$Transcription: \quad G_1^{ab} \xrightarrow{k7} G_1^{ab} + Cre \tag{7}$$

$$Degradation(disappearance): \quad Cre \xrightarrow{k8} \phi \tag{8}$$

$$Degradation(disappearance): \quad A \xrightarrow{k9} \phi \tag{9}$$

$$Degradation(disappearance): \quad B \xrightarrow{k10} \phi \tag{10}$$

$$Creation(appearance): \quad \phi \xrightarrow{k11} A \tag{11}$$

$$Creation(appearance): \quad \phi \xrightarrow{k12} B \tag{12}$$

$$Plasmidtransformation(deletion): \quad Cre + pmd_i \xrightarrow{k13} pmd \tag{13}$$

$$Transcription(constitutive): \quad pmd_i \xrightarrow{k14} pmd_i + F2 \tag{14}$$

$$Activation/deactivation: \quad G_2 + F2 \underset{k_{16}}{\overset{k_{15}}{\rightleftharpoons}} G_2^{F2} \tag{15}$$

$$Transcription: \quad G_2^{F2} \xrightarrow{k17} G_2^{F2} + GFP \tag{16}$$

$$Degradation(disappearance): \quad F2 \xrightarrow{k18} \phi \tag{17}$$

$$Degradation(disappearance): \quad GFP \xrightarrow{k19} \phi \tag{18}$$

All reactions are making use of the rate values shown in table 1. These are the values used for the simulation results shown in Figure 2 and Figure 3 as well as in Figure S1, Figure S2 and Figure S3.

Table 1: **Parameters used in the simulation of the single** ***wire***

Parameter	Meaning	Value
$k_1, k_2, k_3, k_4, k_{15}$	Binding rates	0.1 molecules^{-1} min^{-1}
$k_{-1}, k_{-2}, k_{-3}, k_{-4}, k_{16}$	Unbinding rates	20 min^{-1}
k_5, k_6, k_{14}	Transcription rates	100 min^{-1}
k_7, k_{17}	Transcription rates	150 min^{-1}
k_8, k_9, k_{10}, k_{18}	Degradation rates	0.05 min^{-1}
k_{19}	Degradation rate of GFP	0.07 min^{-1}
k_{11}, k_{12}	Entry rates	500 molecules min^{-1}
k_{13}	Recombination rate	0.02 x 10^{-3} molecules^{-1} min^{-1}

XOR simulation: first NOR gate ("NOR_1")

Reactions from 19 to 38 describe the behaviour of the circuit pictured in Figure 4C (the *program* of the cell strain "NOR_1"). The elements pmd_{F1} and pmd_{F2} make reference to the plasmid (Figure 4E) when the promoter is *pointing towards* gene *F1* or gene *F2* respectively. For simplicity, the expression product of genes $G1$ and $G1^*$ (which share upstream promoter) are transcribed using the same rates (from 23 to 25) so that $G1^*$ is excluded from the model.

$$Activation/deactivation: \quad A + G_1 \underset{k_{-1}}{\overset{k_1}{\rightleftharpoons}} G_1^a \tag{19}$$

$$Activation/deactivation: \quad B + G_1 \underset{k_{-2}}{\overset{k_2}{\rightleftharpoons}} G_1^b \tag{20}$$

$$Upgrade/downgrade\ transcription: \quad B + G_1^a \underset{k_{-3}}{\overset{k_3}{\rightleftharpoons}} G_1^{ab} \tag{21}$$

$$Upgrade/downgrade\ transcription: \quad A + G_1^b \underset{k_{-4}}{\overset{k_4}{\rightleftharpoons}} G_1^{ab} \tag{22}$$

$$Transcription: \quad G_1^a \xrightarrow{k5} G_1^a + fimE + X \tag{23}$$

$$Transcription: \quad G_1^b \xrightarrow{k6} G_1^b + fimE + X \tag{24}$$

$$Transcription: \quad G_1^{ab} \xrightarrow{k7} G_1^{ab} + fimE + X \tag{25}$$

$$Degradation(disappearance): \quad fimB \xrightarrow{k8} \phi \tag{26}$$

$$Degradation(disappearance): \quad fimE \xrightarrow{k8} \phi \tag{27}$$

$$Degradation(disappearance): \quad A \xrightarrow{k9} \phi \tag{28}$$

$$Degradation(disappearance): \quad B \xrightarrow{k10} \phi \tag{29}$$

$$Creation(appearance): \quad \phi \xrightarrow{k11} A \tag{30}$$

$$Creation(appearance): \quad \phi \xrightarrow{k12} B \tag{31}$$

$$Degradation(disappearance): \quad X \xrightarrow{k20} \phi \tag{32}$$

$$Transcription: \quad G_2 \xrightarrow{k21} G_2 + fimB \tag{33}$$

$$Dectivation/activation: \quad G_2 + X \underset{k_{-22}}{\overset{k_{22}}{\rightleftharpoons}} G_2^X \tag{34}$$

$$Plasmidtransformation(inversion): \quad fimB + pmd_{F1} \xrightarrow{k23} pmd_{F2} \tag{35}$$

$$Plasmidtransformation(inversion): \quad fimB + pmd_{F2} \xrightarrow{k24} pmd_{F1} \tag{36}$$

$$Plasmidtransformation(inversion):\quad fimE + pmd_{F1} \xrightarrow{k25} pmd_{F2} \tag{37}$$

$$Plasmidtransformation(inversion):\quad fimE + pmd_{F2} \xrightarrow{k26} pmd_{F2} \tag{38}$$

Table 2: **Parameters used in the simulation of NOR_1**

Parameter	Meaning	Value
$k_1, k_2, k_3, k_4, k_{22}$	Binding rates	0.1 molecules^{-1} min^{-1}
$k_{-1}, k_{-2}, k_{-3}, k_{-4}$	Unbinding rates	20 min^{-1}
k_{-22}	Unbinding rate	10 min^{-1}
k_5, k_6	Transcription rates	100 min^{-1}
k_7, k_{21}	Transcription rates	150 min^{-1}
k_8, k_9, k_{10}, k_{20}	Degradation rates	0.05 min^{-1}
k_{19}	Degradation rate of GFP	0.07 min^{-1}
k_{11}, k_{12}	Entry rates	500 molecules min^{-1}
$k_{23}, k_{24}, k_{25}, k_{26}$	Inversion rates	0.02 x 10^{-3} molecules^{-1} min^{-1}

The rate values used are shown in table 2. These values are used to obtain the simulation results of Figure 5.

XOR simulation: second NOR gate ("NOR_2")

The "NOR_2" gate of the XOR example is, basically, the combination of both the *sender* and the *receiver* cells in Figure 1B, as its circuit shows in Figure 4D. The difference relies on the four different plasmid configurations we can have now (instead of the two possibilities in Figure 1B): pmd_{F2}^{F1}, pmd^{F1}, pmd_{F2} (pmd_i in Figure 1B) and *pmd*. Thus, this simulation uses the rates from 1 to 18 with the next particularities:

We must add these new rates:

$$Plasmidtransformation(deletion):\quad Cre + pmd_{F2}^{F1} \xrightarrow{k27} pmd^{F1} \tag{39}$$

$$Transcription(constitutive):\quad pmd^{F1} \xrightarrow{k28} pmd^{F1} + F1 \tag{40}$$

$$Transcription(constitutive):\quad pmd_{F2}^{F1} \xrightarrow{k29} pmd_{F2}^{F1} + F1 \tag{41}$$

Also, the rate 10 has to be removed, as the cell will not *read* input B (thus, we substitute B by *F1* and G_1^B by G_1^{F1}). The rates of Table 1 are used to obtain the results of Figure S4 (adding $K_{28} = K_{29} = 150$ min^{-1} and $K_{27} = 0.02$ x 10^{-3} molecules^{-1} min^{-1}).

Symmetrical reactions would control the cell strain NOR_3 but taking into account that those cells do not *read* input A instead.

XOR simulation: full study

The full simulation make use of all reactions (1 to 41) and the rates shown in table 3. Each set of equations is *placed* inside its corresponding cell.

Depending on the logic case being studied (0-0, 0-1, 1-0 and 1-1), the inputs of the system (A and B) will be either "0" (0 molecules) or "1" (1000 molecules). The initial conditions for the rest of the elements are: G_{1_0}=1, $G_{1_0}^a$=0, $G_{1_0}^b$=0, $G_{1_0}^{ab} = 0$, G_{2_0}=1, $G_{2_0}^X$=0, $fimB_0$=0, $fimE_0$=0, X_0=0, pmd_{F2_0}=0, $F1_0$=0, Cre_0=0, pmd_0=0, pmd_0^{F1}=0, X_0=0, $GFP_0 = 0$, $pmd_{F2_0}^{F1}$=cp_number. Where

Table 3: **Parameters used in the full XOR simulation**

Parameter	Meaning	Value
$k_1, k_2, k_3, k_4, k_{15}, k_{22}$	Binding rates	0.1 molecules^{-1} min^{-1}
$k_{-1}, k_{-2}, k_{-3}, k_{-4}, k_{16}$	Unbinding rates	10 min^{-1}
k_{-22}	Unbinding rate	5 min^{-1}
k_5, k_6, k_{17}	Transcription rates	150 min^{-1}
k_{14}, k_{21}	Transcription rates	100 min^{-1}
k_7, k_{28}, k_{29}	Transcription rates	200 min^{-1}
$k_8, k_9, k_{10}, k_{18}, k_{20}$	Degradation rates	0.05 min^{-1}
k_{19}	Degradation rate of GFP	0.12 min^{-1}
k_{13}, k_{27}	Recombination rate	0.02 x 10^{-2} molecules^{-1} min^{-1}
$k_{23}, k_{24}, k_{25}, k_{26}$	Inversion rates	0.02 x 10^{-2} molecules^{-1} min^{-1}

"cp_number (copy number) is the number of plasmids that are *allowed* in a cell (value stated in figure captions).

The previous values are used to obtain the simulation results of Figure 6 and Figure S5.
